# The Microbial Quality of Commercial Chopped Romaine Lettuce Before and After the “Use By” Date

**DOI:** 10.3389/fmicb.2022.850720

**Published:** 2022-04-11

**Authors:** Chao Liao, Luxin Wang

**Affiliations:** Department of Food Science and Technology, University of California, Davis, Davis, CA, United States

**Keywords:** Romaine lettuce, “Use By” date, microbial food quality, plate counting, 16S rRNA gene sequencing, bacterial communities

## Abstract

In the United States, due to the limited information about the microbial quality and safety of fresh produce after the labeled open dates, unnecessary discarding of fresh produce in good conditions and food loss have been caused. The aim of this study was to address this knowledge gap and evaluate the microbial quality of commercial chopped Romaine lettuce (RL) on the “Use By” dates (UBD) and 5 days after the “Use By” dates (UBD5). The microbial quality was evaluated using culture-dependent and culture-independent methods. Three brands of RL samples, from early and late harvest seasons, were purchased from local grocery stores and stored at 4°C until 5 days after their UBD. On the UBD and UBD5, bagged lettuce was opened, homogenized, diluted, and plated onto plate count agar and anaerobic agar to obtain total aerobic plate counts (APC) and total anaerobic plate counts (AnPC). For the culture-independent method, DNA was extracted from each sample homogenate and used for 16S rRNA gene sequencing. The culture-dependent results showed that there was no significant change in APC or AnPC between UBD and UBD5 samples. The APC and AnPC ranged from 5.71 ± 0.74 to 7.89 ± 0.10 Log CFU/g and 1.75 ± 0.08 to 7.32 ± 0.61 Log CFU/g, respectively. No significant difference in alpha diversity, based on observed features and Shannon index values, was detected between UBD and UBD5 samples using 16S rRNA sequencing. Similarly, no difference was observed in beta diversity based on the Jaccard distance matrixes and the weighted Unifrac distance matrixes. Taxonomic analysis revealed 128 genera in all RL samples. The top five genera were *Pseudomonas* (with relative abundance ranging from 16.47 to 92.72%), *Serratia* (0–52.35%), *Weissella* (0–42.42%), *Pantoea* (0.17–21.33%), and *Lactococcus* (0–24.30%). The differential abundance analysis based on the ANCOM test showed that no bacteria were detected to have significantly differential abundance in RL between UBD and UBD5. In summary, both the culture-dependent and culture-independent results showed that there was no significant difference in the microbial quality of RL before and shortly after the UBD.

## Introduction

Although fruits and leafy greens are essential components of a healthy diet, they are also one of the most wasted foods, with up to 63% of fresh fruits and 70% of leafy greens wasted (Mijares et al., 2021). The United States Department of Agriculture (USDA) estimated that approximately 31% of the 430 billion pounds of food produced in 2010 (valued at ca. $161.6 billion) was lost and not available for human consumption at the retail and consumer levels (Buzby et al., 2014). Vegetables (19%) ranked second on the list of food groups with high loss rates (Buzby et al., 2014). In the USDA and Environmental Protection Agency (2015) announced that the US 2030 Food Loss and Waste Reduction goal was to reduce 50% of food loss and waste by the year 2030 (Environmental Protection Agency, 2015; United States Department of Agriculture, 2015). There are many causes and drivers for food loss and waste at the retail level; consumers’ confusion over “Use By” and “Best Before” dates and other date labeling is one of them (Newsome et al., 2014; Buzby et al., 2015). Date labeling has been practiced for decades, and its main aims include informing stock rotation at retail and facilitating potential recalls and traceability. However, the terminologies used vary widely around the world (Newsome et al., 2014).

Such variations in date labeling terms have contributed to substantial misunderstanding by the industry and consumers and led to significant unnecessary food loss and waste, especially in the United States (Newsome et al., 2014). Recent studies showed that approximately 37% of American consumers usually discarded fresh produce when the open date is past even when the product had no quality and safety problems (Leib et al., 2016; Wilson et al., 2017). In the United States, three date labels, namely, “Best if Use By” or “Best Before,” “Use By,” and “Sell By,” are the most applied for food products (Wilson et al., 2017). Tsiros and Heilman (2005) summarized the three commonly used label phrases: (1) “Best before,” which indicates the date after which a product loses the best quality for consumption. (2) “Use By,” indicating the date after which a product is no longer remaining sufficient quality and should not be consumed, but not necessarily associated with food safety. (3) “Sell by,” suggesting the last day on which a product should be sold (Tsiros and Heilman, 2005; Wilson et al., 2017). Among these three phrases, “Use By” generates the greatest value of predicted waste (Wilson et al., 2017).

In contrast to the United States, Regulation No. 1169/2011 of the European Parliament and the Council of the European Union (EU) mandates defined the use of date of minimum durability or best before date, and “Use By” date (European Commission, 2011). Foods that are highly perishable and likely to constitute an immediate danger to human health should carry a “Use By” date (UBD), after which date the “food shall be deemed unsafe” (European Food Safety Authority Panel on Biological Hazards [BIOHAZ] et al., 2020). In Australia and New Zealand, date labeling with a best before date or “Use By” date is required for most packaged foods with a shelf life of less than 2 years (Australian Government, 2012). “Best before” date indicates “the last date on which you can expect a food to retain all of its quality attributes, provided it has been stored according to any stated storage conditions and the package is unopened” (Food Standards Australia New Zealand, 2013). UBD is “the last date on which the food may be eaten safely, provided it has been stored according to any stated storage conditions and the package is unopened” and the food should not be eaten due to the health and safety reasons after the date (Food Standards Australia New Zealand, 2013).

Microorganisms play significant roles in food spoilage and loss (Lorenzo et al., 2018). The microbiological quality and safety of fresh produce are determined by factors from pre-harvesting to post-harvest. To better characterize the microorganisms present in fresh produce during storage, a number of research projects have applied culture-dependent and culture-independent methods to investigate the microbiological quality of fresh produce during storage (Jeddi et al., 2014; Berthold-Pluta et al., 2017; Cruz et al., 2019; Arienzo et al., 2020). For instance, Jeddi et al. (2014) enumerated the total aerobic bacteria present in 116 samples of fresh-cut vegetables, ready-to-eat salads, and wheat and mung bean sprouts before their open dates. The total aerobic mesophilic bacterial counts were 5.3–7.5, 5.5–7.4, 5.5–8.4, and 6.4–8.5 Log CFU/g, respectively for fresh-cut vegetable, ready-to-eat salads, and wheat and mung bean sprouts, respectively. Similarly, Berthold-Pluta et al. (2017) reported that the total aerobic mesophilic bacterial counts in lettuces, sprouts, and non-pasteurized fruits and vegetables during cold storage (below 4^°^C) before the “Best Before” dates were in ranges of 5.6–7.6, 6.8–8.4, and 2.9–7.7 Log CFU/g, respectively.

By using 16S rRNA sequencing, Leff and Fierer (2013) profiled the bacterial communities present on 11 produce varieties purchased from grocery stores, such as apples, grapes, lettuce, mushrooms, peaches, peppers, spinach, strawberries, tomatoes, alfalfa sprouts, and mung bean sprouts. Their results showed that the bacterial communities associated with each product type differed remarkably from each other, but certain produce types appeared to share more similar bacterial communities, such as sprouts, spinach, lettuce, tomatoes, peppers, and strawberries. Keshri et al. (2019) determined the dynamics of the bacterial communities of fresh alfalfa and mung bean sprouts right after purchase and every week until the end of their shelf-life. They found that both sprout types contained spoilage-related bacteria, *Pseudomonas* and *Pantoea*, at the time of purchase. The abundance of *Pseudomonas* increased in alfalfa after 3 weeks of cold storage at 4^°^C, while *Pantoea* dominated in mung bean sprouts after 2 weeks of storage at the same temperature. While previous studies provide important insights into the microbial population composition and potential changes during the shelf life of fresh produce, most of their sampling ended on the UBD. No information is available about bacterial populations present in fresh produce after the UBD. However, such information is very much needed for the industry as well as consumers to be better informed about what happens to the microbial quality after the UBD.

To fill the above knowledge gap, the objectives of this study were to investigate the bacterial populations of three brands of bagged chopped Romaine lettuce (RL) purchased during different seasons (early season *vs*. late season) and then evaluate the microbial quality of RL on the UBD and 5 days after the “Use By” dates (UBD5), using both culture-dependent and culture-independent methods.

## Materials and Methods

### Romaine Lettuce Samples

Three brands (Brands A, B, and C) of commercial chopped RL products were purchased from local grocery stores during the early (September–October) and late (March–April) harvest seasons (Williams et al., 2013). Three batches of RL for each season were purchased for the triplicate experiment. Upon arrival at the laboratory, all samples were stored at 4^°^C and sampled on their labeled UBD and UBD5. Photos were also taken for these samples on each sampling day (Supplementary Figure 1). As shown in Supplementary Figure 1, no significant visual differences were observed between UBD and UBD5.

### Culture-Based Analysis of the Microbial Quality of Romaine Lettuce

At each sampling point presenting different shelf lives (UBD or UBD5), 80 g of RL was taken from each bag and added in a 55 oz Whirl-Pak filter bag (Nasco, Fort Atkinson, WI, United States) with 320 ml of phosphate-buffered saline (PBS, pH 7.4). The filter bag with RL samples was homogenized by using the Smasher™ Lab Blender (AES-Chemunix, Bruz, France) for 120 s at the fast speed (620 strokes/min). One milliliter of the homogenate was serially diluted in 9 ml of PBS in 15 ml Falcon tubes (VWR, Atlanta, GA, United States), and four 100 μl of each dilute was plated on two plate count agar (PCA, BD Biosciences, Sparks, MD, United States) and two anaerobic agar (AA, BD Biosciences). PCA plates were incubated at 37^°^C for 48 h before colony enumeration. Anaerobic agar count medium supplemented with 2 mg/L of Methylene blue as the indicator of oxygen levels (light blue color for the presence of oxygen and yellowish color for the absence of oxygen) was used for culturing and enumerating anaerobes. Plated AA agar was incubated in Mitsubishi Gas Chemical (MGC) AnaeroPack-Jars with two MGC AnaeroPack-Anaero packs (Mitsubishi Gas Chemical Co., Tokyo, Japan) in each jar at 37^°^C for 48 h before enumeration (Liao and Wang, 2021). The generated total aerobic plate counts (APC) and total anaerobic plate counts (AnPC) were calculated and expressed as mean ± standard deviation (*SD*) Log CFU/g. The limit of detection of this plating method was 1.7 Log CFU/g of lettuce.

### DNA Extraction and 16S rRNA Gene Sequencing

From each homogenate prepared above at each sampling point, 10 ml of the homogenate was transferred into a 15 ml Falcon tube (VWR, Atlanta, GA, United States) and centrifuged at 3,000 × *g* for 10 min at 4^°^C by using the Eppendorf Centrifuge 5810 R (Eppendorf, Hamburg, Germany) to collect the cell pellets. Each cell pellet was then washed twice by using 10 ml of PBS *via* centrifugation. Each washed pellet was then re-suspended with 1 ml of PBS and transferred into a 1.5 ml microcentrifuge tube (VWR, Atlanta, GA, United States). DNA was extracted from these pellets by using the DNeasy Powersoil kit (Qiagen, Gaithersburg, MD, United States) following the manufacturer’s instructions. A total of 36 DNA samples were extracted and stored at −80^°^C for the subsequent 16S rRNA sequencing process. The library construction was carried out based on the amplification of V3–V4 region of the 16S rRNA gene (341F: 5′-CCTACGGGNGGCWGCAG-3′ and 785R: 5′-GACTACHVGGGTATCTAATCC-3′). Every PCR amplicon was tagged with forward and reverse barcodes (7 bp) (Sinclair et al., 2015; Liao and Wang, 2021). The sequencing was performed using the Illumina^®^ MiSeq instrument with the MiSeq Reagent Kit v3 (Illumina, San Diego, CA, United States) to produce 2 × 300 bp paired-end reads. Library preparation and sequencing were conducted at the HudsonAlpha Genomic Service Laboratory (Huntsville, AL, United States).

### Microbiome Data Analysis

The 16S rRNA gene sequencing data were processed using the Quantitative Insights Into Microbial Ecology (QIIME 2 version 2021.4) pipeline (Bolyen et al., 2019). De-multiplexed sequences were obtained from the Illumina BaseSpace platform by assigning reads to each sample based on sample-specific barcodes. For quality control, barcodes and primers were trimmed from the raw sequences using a q2-cutadapt plugin (Martin, 2011), followed by the denoising process to filter out the noisy reads, remove chimeric and singleton sequences, join denoised pair-end sequences, and cluster sequences using q2-dada2 plugin (Callahan et al., 2016). The principle of the DADA2 plugin in the QIIME 2 pipeline (q2-dada2 plugin) was based on the interactive quality plots of the Phred score as a function of each base in paired-end sequences (Supplementary Figure 2). The base position after which the median Phred quality scores of bases dropping below 30 was applied as the cut-off point for truncation of sequences in the same length (Estaki et al., 2020). With parameters “–p-trunc-len-f” (truncating length for forward reads), “–p-trunc-len-r” (truncating length for reverse reads), “–p-max-ee-f” (the number of maximum expected errors for forward reads), and “–p-max-ee-r” (the number of maximum expected errors for reverse reads) set as 260, 185, 2, and 4, respectively, approximately 62% of the sequences with median Pred quality score greater than 30 were kept for the downstream sequence analysis (Supplementary Table 1).

In this step, the amplicon sequence variants (ASVs) were clustered based on 100% of sequence similarity (Callahan et al., 2017), and the feature table of ASVs with frequency and feature data of representative ASVs was generated. A phylogenetic tree was constructed based on the feature data by using the align-to-tree-mafft-fasttree plugin (Katoh and Standley, 2013). Diversity analyses were conducted by using the core-metrics-phylogenetic plugin (Caporaso et al., 2010) with the parameter of sampling depth set as the minimum library size across all samples (80,938 reads). Alpha diversity was evaluated based on the observed features (ASVs) and Shannon index values (Kõiv et al., 2019). The beta diversity was evaluated by using the Jaccard distance matrix and the weighted Unifrac distance matrix (Lozupone et al., 2011; Edgar, 2017). Beta diversity was then visualized *via* the two-dimensional principal coordinate analysis (PCoA) plots. Taxonomic analysis was conducted at the genus level using the classify-sklearn plugin (Bokulich et al., 2018a,b), which employed the pre-trained Naive Bayes classifier based on SILVA 138 small subunit rRNA^1^ database (Quast et al., 2013; Bokulich et al., 2018a).

### Statistical Analyses

All experiment trials were carried out in three independent replicates. Analysis of variance (ANOVA) and Tukey’s Honestly Significant Difference (HSD) test (Abdi and Williams, 2010) were applied to analyze the mean differences of bacterial amounts (APC or AnPC) obtained from RL of different brands (Brands A, B, and C) purchased during different seasons (early *vs*. late season), as well as at different points in their shelf lives (UBD *vs*. UBD5). The non-parametric Wilcoxon rank sum test and the Kruskal–Wallis test were applied to test the difference between alpha diversities among groups (Xia and Sun, 2017). The permutational multivariate analysis of variance (PERMANOVA) (Anderson, 2014) was used for analyzing the difference of beta diversities and the impact of shelf life, brand, and harvest season on beta diversity. Analysis of composition of microbiomes (ANCOM) (Mandal et al., 2015) was applied to identify bacteria that had significantly differential abundance between groups when samples were grouped and compared by shelf life, brands, or harvest seasons. The identification of these bacteria was based on the calculation of the centered log ratio (clr) F statistic and the W statistic. The clr F statistic measured the differences in effect size between groups based on the clr transformed bacterial abundance data, while the W statistic represented the number of times the log-ratio of a taxon with every other taxon being tested was identified to be significantly different across groups (Stevens et al., 2019). All the above analyses were performed using R scripts (version 4.1.0). Differences were considered statistically significant when the probability (*p*) value was less than 0.05.

## Results

### Changes in Aerobic Plate Counts and Anaerobic Plate Counts in Romaine Lettuce on the UBD and UBD5

**FIGURE 1:**
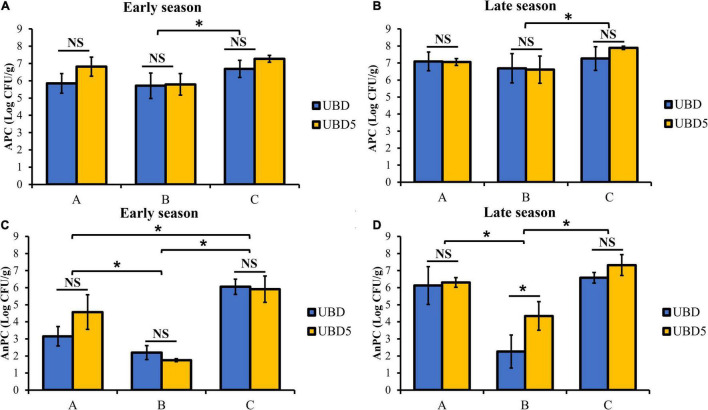
Total aerobic plate count (APC, panels **A,B**) and anaerobic bacterial counts (AnPC, panels **C,D**) of three brands of RL purchased in the early and late seasons and analyzed on the their “Use By” dates (UBD) and 5 days after the “Use By” dates (UBD5). *Represents significant differences of APC or AnPC between RL samples of three brands or between UBD and UBD5 (*p* < 0.05).

As shown in Figure 1, the APC of RL samples ranged from 5.71 ± 0.74 to 7.27 ± 0.20 Log CFU/g when purchased and sampled in the early season (Figure 1A) and ranged from 6.61 ± 0.79 to 7.89 ± 0.10 Log CFU/g in the late season (Figure 1B). At each harvest season, no difference in APC was detected between samples plated on the UBD and UBD5. When comparing the APC among three brands, brand C (6.98 ± 0.46 Log CFU/g in the early season and 7.57 ± 0.56 Log CFU/g in the late season) had statistically higher APC counts than brand B (5.75 ± 0.61 Log CFU/g in the early season and 6.65 ± 0.74 Log CFU/g in the late season). The AnPC of RL ranged from 1.75 ± 0.08 to 6.05 ± 0.44 Log CFU/g in samples from the early season (Figure 1C) and 2.26 ± 0.97 to 7.32 ± 0.61 Log CFU/g in samples from the late-season (Figure 1D). No significant difference in RL AnPC was detected between UBD and UBD5 except for brand B when samples were tested in the late season. The AnPC of brand B was 2.26 ± 0.97 Log CFU/g on UBD and was 4.34 ± 0.84 Log CFU/g on UBD5 when sampled in the late season. For samples purchased and analyzed in the early season, regardless of shelf-life, brand C had the highest AnPC among all three brands (5.98 ± 0.57 Log CFU/g), while brand B showed the lowest AnPC (1.97 ± 0.36 Log CFU/g). For samples from the late harvest season, brand B had the lowest AnPC (3.30 ± 1.40 Log CFU/g) among all three brands.

### Amplicon Sequence Variant Identification and the Diversity Analyses of Bacterial Communities

A total of 36 DNA samples were sequenced using 16S rRNA sequencing. With the denoising process in QIIME 2 pipeline, 1,179 ASVs were identified from the total 6,051,758 sequences (Supplementary Table 1). The minimum sequence frequency, 80,938, was chosen for rarefying the reads across all samples to avoid false positive detection (Saary et al., 2017; Supplementary Figure 3). The impacts of shelf life (UBD and UBD5), brand (brands A, B, and C), and harvest season (early and late season) on the alpha and beta diversity of bacterial communities were analyzed by using the QIIME 2 pipeline.

**FIGURE 2:**
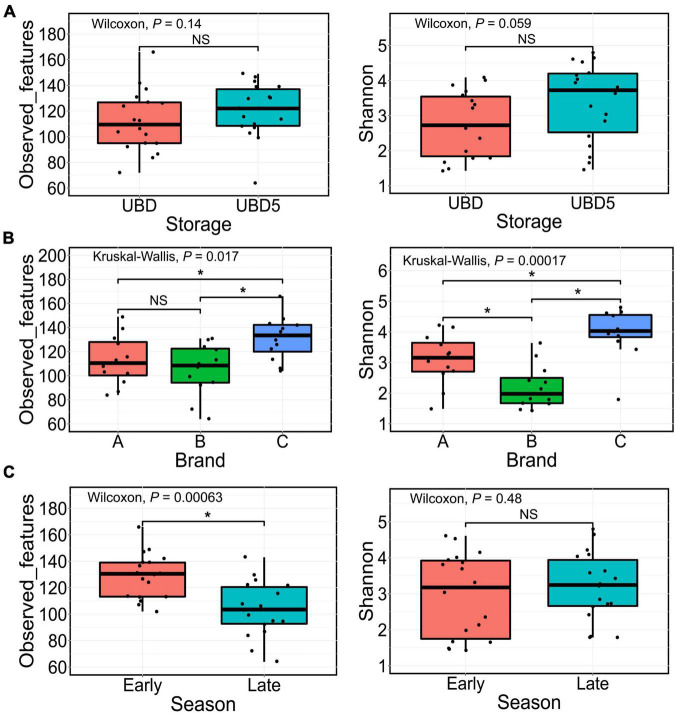
Comparison of the alpha diversity based on the observed features (left) or the Shannon index values (right) of microbial populations present in RL between UBD and UBD5 **(A)**, among three brands **(B)**, and between two seasons **(C)**. The Wilcoxon rank test was used for pairwise comparison while the Kruskal–Wallis test was applied for comparisons among three groups. *Represents significant differences between groups (*p* < 0.05). NS means no significant difference.

The alpha diversity of bacterial communities was evaluated by measuring observed features (ASVs) and the Shannon index values (Figure 2). As shown in Figure 2A, the numbers of the observed features (left) and the Shannon index values (right) showed no difference between UBD and UBD5 (*p* > 0.05). When comparing alpha diversity among the three brands, brand C had 131 ± 18 observed features (Figure 2B left), which was remarkably higher than the observed features from brands A and B (*P* = 0.043 when comparing brand A and brand C; *p* = 0.0072 when comparing brands B and C based on the Wilcoxon rank sum test). No difference of observed features was detected between brands A and C. Brand C samples also had the highest Shannon index value of 3.95 ± 0.80, compared with brand A (3.09 ± 0.82, *p* = 0.01) and brand B (2.19 ± 0.71, *p* = 5 × 10^–5^) (Figure 2B, right). Brand A had a higher Shannon index value than brand B (*p* = 0.012). The Kruskal–Wallis test showed that the factor of “brand” pronouncedly impacted the observed features (*p* = 0.017) and Shannon index values (*p* = 1.7 × 10^–4^) of RL bacterial communities. When focusing on the impact of harvest seasons, early harvest-season RL had higher observed features (129 ± 17) than late-season RL (104 ± 21) (*p* = 6.3 × 10^–4^, Figure 2C left). However, such impact was not detected when focusing on the Shannon index values (Figure 2C right).

Changes in the alpha diversity of bacterial communities in each brand of RL between UBD and UBD5 were also studied (Supplementary Figure 4). It can be seen that the brand A RL had a significant increase of observed feature numbers from UBD to UBD5, while no difference of Shannon index values was observed between RL on the UBD and UBD5. Brand B RL had no changes in neither observed feature number nor Shannon index value from UBD to UBD5. For the brand C RL, Shannon index value had a remarkable increase from UBD to UBD5, while no difference of observed feature numbers was detected between UBD and UBD5.

**FIGURE 3:**
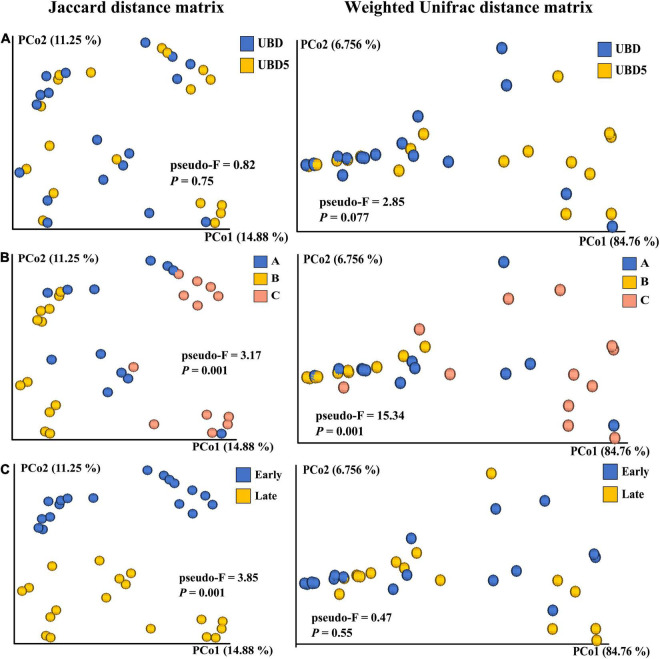
Comparison of the beta diversity based on the Jaccard distance matrix (left) and the weight Unifrac distance matrix (right) of microbial communities present in RL between UBD and UBD5 **(A)**, among three brands **(B)**, or between different seasons **(C)**. The PERMANOVA test was applied to analyze the differences in each group when samples were grouped by shelf life, brand, or seasons. “pseudo-F” values are measures of the effect size, indicating the differences within each group of RL. The *p*-values of less than 0.05 indicate that the factor of shelf life, brand, or season significantly impacts the beta diversity.

The beta diversity of the RL bacterial community was calculated based on the Jaccard distance matrix and the weighted Unifrac distance matrix. PCoA was used to visualize the beta diversity of bacterial communities in RL samples grouped by shelf life, brands, or seasons. The PERMANOVA test was employed to statistically evaluate the impacts of shelf life, brands, or seasons on the beta diversity. In general, PCoA plots based on the Jaccard distance matrix (Figure 3 left) showed that the principal coordinates 1 and 2 explained 14.88 and 11.25% of variances, respectively, and PCoA plots based on the weighted Unifrac distance (Figure 3 right) showed that the PCo1 and PCo2 explained 84.76 and 6.76% of variances, respectively, suggesting that the weighted Unifrac distance matrix captured more commensal bacteria variances than the Jaccard distance matrix, as illustrated on the two-dimensional PCoA plots. No obvious visual separation was observed between UBD and UBD5 samples. This result was further confirmed by using the PERMANOVA test with *p*-values of 0.767 and 0.077 for the Jaccard distance-based comparison and the weighted Unifrac-based comparison, respectively. The factor of “brand,” on the other hand, more significantly impacted the beta diversity of the RL bacterial community, as the PCoA plots based on the Jaccard distance matrix and the weighted Unifrac distance both showed clear visual separation among the three brands. The PERMANOVA test confirmed the significant impact of the factor “brand” on the beta diversity of the RL bacterial community by generating *p*-values of 0.001 for both the Jaccard distance-based comparison and the weighted Unifrac distance-based comparison. When analyzing the impact of harvest season on beta diversity, analyses based on the Jaccard distance was different from the results obtained based on the weighted Unifrac. The PCoA plot presented a clear visual separation between early and late season samples when using the Jaccard distance-based comparison (*p*-value = 0.01 in the PEMANOVA test), while no separation was observed from the PCoA plot based on the weighted Unifrac distance matrix (*p*-value = 0.55 in the PEMANOVA test).

### Taxonomic Analysis of Commensal Bacteria in Romaine Lettuce

**FIGURE 4:**
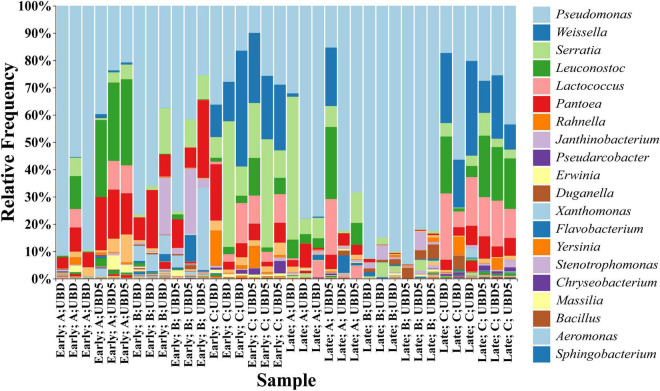
Relative abundance of the top 20 bacteria genera identified from all three brands of RL purchased from two harvest seasons at UBD and UBD5. “UBD” and “UBD5” mean “Use By” dates and 5 days after the UBD. “A,” “B,” and “C” stand for three brands and “Early” and “Late” represent the early and late harvest season.

The taxonomic analysis of commensal bacteria identified 128 genera across all RL samples based on the SILVA database. The top five genera were *Pseudomonas* (with relative abundances ranging from 9.95 to 94.73%), *Weissella* (0–42.42%), *Serratia* (0–52.35%), *Leuconostoc* (0–31.56%), and *Lactococcus* (0–24.30%) (Figure 4). When focusing on shelf life, the top five genera identified from the UBD samples were *Pseudomonas* (16.47–92.72%), *Serratia* (0–52.35%), *Weissella* (0–42.42%), *Pantoea* (0.17–21.33%), and *Lactococcus* (0–24.30%), while the top five genera identified on UBD5 were *Pseudomonas* (9.95–94.73%), *Pantoea* (0–31.56%), *Leuconostoc* (0.23–28.50%), *Serratia* (0–25.66%), and *Weissella* (0.10–39.62%).

When focusing on different brands, the top five genera identified in brand A samples were *Pseudomonas* (15.32–91.62%), *Leuconostoc* (0.01–31.56%), *Serratia* (0–52.35%), *Pantoea* (0.60–19.48%), and *Lactococcus* (0.2–0.42%); the top five genera identified in brand B were *Pseudomonas* (25.46–94.73%), *Pantoea* (0.17–28.50%), *Janthinobacterium* (0.47–24.01%), *Xanthomonas* (0–30.26%), and *Serratia* (0.05–16.77%); while the top five genera identified in brand C were *Pseudomonas* (9.95–56.41%), *Weissella* (9.21–42.42%), *Serratia* (0.28–46.06%), *Lactococcus* (0.94–24.30%), and *Leuconostoc* (1.14–22.52%). When grouping the samples by harvest seasons, the top five genera identified in the early season were *Pseudomonas* (9.95–91.62%), *Pantoea* (1.64–28.50%), *Serratia* (0–46.06%), *Weissella* (0–42.42%), and *Leuconostoc* (0–31.56%), while the top five genera identified in the late season were *Pseudomonas* (15.33–94.73%), *Weissella* (0–34.69%), *Leuconostoc* (0–26.41%), *Serratia* (0.05–52.35%), and *Lactococcus* (0–24.30%).

### Differential Abundance Analysis of Commensal Bacteria in Romaine Lettuce Based on Shelf Life, Brand, and Harvest Season

**FIGURE 5:**
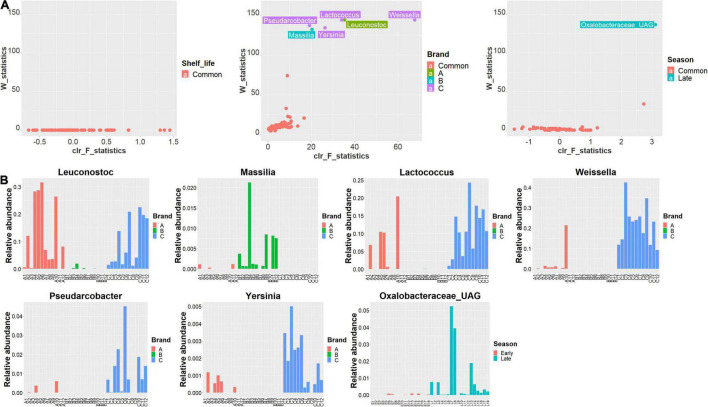
**(A)** Volcano plots of bacteria, at the genus level, identified to have different abundances when RL are grouped and compared based on their shelf-life (left), brand (middle), and harvest season (right) with the ANCOM test. The abundance differences of bacteria were evaluated by the clr F-statistic and W-statistic. Bacteria with no different abundance when RL samples were grouped and compared based on shelf life, brand, and season factors are marked in red. Bacteria with significantly higher abundances in the brands A, B, and C RL are marked in green, blue, and purple colors, respectively, when comparing the RL of three brands. Bacteria with significantly higher abundances in the late season RL are marked in blue when comparing RL of different seasons. **(B)** Barplots of the relative abundance changes of *Leuconostoc*, *Massilia*, *Weissella*, *Lactococcus*, *Pseudarcobacter*, *Yersinia* identified as the biomarkers for the three brands of RL and Oxalobacteraceae identified as the biomarker for the late season RL.

To identify the critical features (biomarkers) associated with samples when grouped by shelf life, brand, and harvest season, the ANCOM analysis was employed for the differential abundance analysis of commensal bacteria in RL. Results of ANCOM analysis are shown in Figure 5A. No bacteria were identified to be significantly different in abundance between UBD and UBD5, while six genera were identified to have significantly differential abundance among three brands; these were *Weissella* (W statistics = 144, clr F statistics = 67.7), *Leuconostoc* (W statistics = 144, clr F statistics = 35.3), *Lactococcus* (W statistics = 144, clr F statistics = 33.9), *Pseudarcobacter* (W statistics = 137, clr F statistics = 19.1), *Yersinia* (W statistics = 134, clr F statistics = 26.3), and *Massilia* (W statistics = 132, clr F statistics = 20.3). *Leuconostoc* (RA 0.011–31.56%) had the highest RA in brand A, while *Massilia* (0–2.12%) had the highest abundance in brand B. *Weissella* (9.21–42.42%), *Lactococcus* (RA 0.94–24.30%), *Pseudarcobacter* (0–4.51%), and *Yersinia* (0–0.50%) had the highest RA in brand C. *Lactococcus*, *Leuconostoc*, *Weissella*, *Pseudarcobacter*, and *Yersinia* had the lowest RA in brand B (Figure 5B). When grouping RL samples by harvest seasons, only one unannotated genus (UAG) of Oxalobacteraceae (W statistics = 137, clr F statistics = 3.1) had significantly higher RA in the samples from the late harvest season (0–5.24%) than in the samples from the early harvest season (0–0.083%) (Figure 5B). Based on the ANCOM test, no bacteria in the RL of brands A, B, and C individually could be identified as biomarkers for between UBD and UBD5 samples (Supplementary Figure 4).

## Discussion

This study applied culture-dependent and culture-independent methods to investigate the commensal bacteria present in bagged RL on their UBD and UBD5. The combination of both methods allows us to better characterize the bacterial populations and discover potential changes associated with abundance, diversity, and composition of different bacterial groups. The application of 16S rRNA gene sequencing is advantageous for microbiome profiling and analyzing bacterial community dynamics, as it is culture-independent and relatively unbiased compared to traditional culture methods that rely highly on selective or differentiated media (Grim et al., 2017).

During the quality control step of analyzing the sequence data in the QIIME 2 pipeline, parameters “–p-trunc-len-f,” “–p-trunc-len-r,” “–p-max-ee-f,” and “–p-max-ee-r” were set at 260, 185, 2, and 4, respectively, in the q2-dada2 plugin command. Estaki et al. (2020) recommended the setting of the read length at which the median Phred quality score began to drop below 30 or 20 if the entire read quality was too low. With these settings, the forward reads and reverse reads were truncated at 260 and 185 bp as their median Phred quality score of the next base started to drop below 30 as shown in the interactive quality plots (Supplementary Figure 2). In addition, the forward reads with more than two erroneous bases and reverse reads with more than four erroneous bases were discarded, as the reverse reads had lower Phred quality scores than the forward reads (Supplementary Figure 2). Similar observations have been reported by D’Amore et al. (2016) and Gerasimidis et al. (2016).

As shown in Figure 1, culture-dependent results showed no difference in APC or AnPC of RL between UBD and UBD5, except the AnPC of late-season brand B. The population density of culturable commensal bacteria in the RL on the UBD and UBD5 remained at the same level for aerobic bacteria and anaerobic bacteria, indicating that the short 5-day storage after the UBD had no significant impact on the total culturable bacterial abundance. It can be explained by that bacterial populations on UBD and UBD5 had reached stationary levels under this particular storage condition. The APC of bagged RL ranged from 5.71 ± 0.74 to 7.89 ± 0.10 Log CFU/g and the AnPC ranged from 1.75 ± 0.08 to 7.32 ± 0.61 Log CFU/g. RL samples obtained from the late season had higher APC (*p* = 3.08 × 10.5) and AnPC (*p* = 1.06 × 10.4) than RL samples obtained from the early season. Culturable bacteria counts obtained from this study were consistent with results from previous studies. Aycicek et al. (2006) reported that the outer leaves of RL and iceberg-lettuce samples harbored 3.3–7.4 Log CFU/g of aerobic bacteria. Korir et al. (2016) reported that the mean abundance of total aerobic bacteria was 7.76 Log CFU/g for lettuce. Similarly, Liao and Wang (2021) reported that the total aerobic bacteria in Spring Mix salad samples were at the level of approximately 6.6 Log CFU/g. The differences observed between different seasons were also reported by Williams et al. (2013) in which the abundance of aerobic bacteria in the phyllosphere was found to be lower on RL planted in the early season (June) (approximately 3.8–5.5 Log CFU/g) than on RL planted in the late season (August and October) (approximately 5.0–6.2 Log CFU/g).

The diversity analysis of the 16S rRNA gene sequencing detected no significant difference of alpha diversity in RL between UBD and UBD5, indicating that neither the richness nor the evenness of the RL bacterial communities had changed. Beta diversity based on the Jaccard distance matrix and the weighted Unifrac distance matrix also showed no difference between UBD and UBD5, as visualized on the PCoA plots. The results were further confirmed by the PERMANOVA test. These results indicate that the additional 5-day storage after the UBD has no impact on the diversity of RL bacterial communities. Similar results have been reported by our previous Spring Mix study in which no significant difference in the beta diversity was observed among Spring Mix samples collected at different storage times during 15 days of cold storage at 4^°^C (Liao and Wang, 2021).

The top six identified genera, *Pseudomonas*, *Serratia*, *Weissella*, *Pantoea*, *Lactococcus*, and *Leuconostoc*, were the same across RL samples, but in different orders. The exception was that the top six genera in brand B samples included *Janthinobacterium*, *Xanthomonas*, and *Flavobacterium* instead of *Weissella, Lactococcus*, and *Leuconostoc*. Among the top six genera, *Pseudomonas* consistently dominated the RL bacterial communities with RA ranging from 9.95 to 94.73%. The previous study carried out by (Gu et al., 2018) reported that the *Pseudomonas* species consistently dominated microbial communities across all spinach samples during week-long storage at 4^°^C (up to 34.20%). Keshri et al. (2019) reported that *Pseudomonas* dominated the bacterial communities of alfalfa sprouts throughout 3 weeks of cold storage at 4^°^C, with an RA of greater than 60%. The *Pseudomonas* genus contains a group of species associated with fresh produce spoilage, such as *Pseudomonas fluorescents*, *P. marginalis*, *P. viridiflava*, and *P. chloroaphis*, and is able to outcompete other bacteria in food matrixes (Hibbing et al., 2010; Langsrud et al., 2016). Another potential spoilage genus is *Pantoea* (Bae et al., 2014; Keshri et al., 2019). The study from Bae et al. (2014) and Keshri et al. (2019) reported that the *Pantoea* RA was up to 50.40% in mung bean sprouts after 2 weeks of storage at 4^°^C. A previous study (Leff and Fierer, 2013) also reported that *Pantoea* was highly abundant in pepper (11.5%), spinach (32.5%), and sprouts (57.5%) analyzed right after purchase.

*Weissella*, *Lactococcus*, and *Leuconostoc* have been reported as psychrotrophic lactic acid bacteria (LAB) potentially linked to the spoilage of fresh produce (Säde et al., 2016; Keshri et al., 2019). Pothakos et al. (2014) reported that *Leuconostoc* spp. was the most dominant population in ready-to-eat salads at the end of their shelf-life (7 days) at 4^°^C, which caused the early spoilage of vegetables before the end of shelf-life. Keshri et al. (2019) found that the abundance of *Leuconostoc* increased after 2 weeks of cold storage at 4^°^C in mung bean sprouts, the bacterium is considered to play a role in the spoilage of sprouts. In addition, greater numbers of *Lactococcus* and *Weissella* were reported in Spring Mix salad when being stored at 4^°^C for 15 days (Liao and Wang, 2021). *Serratia* is a plant-associated genus that is a non-pathogenic symbiont that has also been reported to be present at high levels in organic green leafy lettuce with up to 66% RA (Jackson et al., 2013). *Yersinia* (0–0.50%) and *Bacillus* (0–3.0%), two genera containing human pathogens, e.g., *Yersinia enterocolitica* (Cristiano et al., 2021) and *Bacillus cereus* (Chon and Seo, 2021), were also identified in RL. No significant change in their relative abundance was detected from UBD to UBD5 based on the ANCOM test either.

In this study, ANCOM analysis was applied to analyze and identify biomarkers (bacterial genera) with differential RA in RL samples when grouped by different shelf lives (UBD *vs*. UBD5), brands (brands A, B, and C), and harvest seasons (early *vs*. late). For RL on the UBD and UBD5, the ANCOM test showed no biomarker with significantly differential RA between them, suggesting that the composition of bacteria in the RL had no change 5 days past the UBD. When comparing the microbial populations among three brands, the ANCOM test identified that five genera had significantly different RAs among the three brands. Among them, *Leuconostoc*, *Weissella*, *and Lactococcus* contain species associated with food spoilage, such as *Leuconostoc citreum*, *Leuconostoc mesenteroides*, and *Weissella confuse* (Säde et al., 2016). For the RL from early and late seasons, ANCOM identified only one genus, Oxalobacteraceae_UAG (up to 0.80%), was identified as higher RA in the RL from late season compared to that from early season. Other than this genus in a low RA, RL from the two seasons had no difference in composition of bacterial communities, which was also reflected in the above Shannon diversity of the two-seasonal RL. Williams et al. (2013) illustrated that the season-of-planting factor more strongly impacted RL bacterial communities after 4–8 weeks of planting in the field. Specifically, *Pantoea*, *Pseudomonas*, *Erwinia*, and members of Enterobacteriaceae were identified as the most abundant genera in late season plants. *Leuconostoc*, *Lactococcus*, *Bacillus*, and *Exiguobacterium* were predominant in early season plants. By comparison, the weaker impact of the seasonal factor on the diversity and composition of RL bacterial communities in our study could be explained by postharvest processing (e.g., washing, packaging, and cold storage), which might alter the RL bacterial communities and mitigate the seasonal impact.

In the United States, except for infant formula, the U.S. Food and Drug Administration (FDA) does not require manufacturers to display specific open date labeling on their food products (Wilson et al., 2017). Although a few poultry, meat, and egg products under the jurisdiction of USDA need data documentation, no strict guidelines for using the food date terminology on food products have been set by the USDA (Leib et al., 2013; Newsome et al., 2014). The current open-date labels used in food markets are not well regulated, as the “UBD,” “Best By,” and “Sell By” are interchangeably applied by manufacturers (Wilson et al., 2017). This ambiguous food date labeling has fostered confusion about food product safety and quality among consumers, which subsequently causes unnecessary levels of food waste (Wansink and Wright, 2006; Newsome et al., 2014). Evidence showed that consumers waste food products when they are near the open dates perceived for food quality reasons or food safety reasons (Tsiros and Heilman, 2005; Theotokis et al., 2012; Newsome et al., 2014; Wilson et al., 2017).

In January 2017, the Grocery Manufacturers Association (GMA) and Food Marketing Institute (FMI) recommended the use of two introductory phrases for food date labeling, such as “Best If Used By” and “Use By.” They recommended that “Best If Used By” is applied to “indicate to the consumer that, after a specified date, the product may not taste or perform as expected but is safe to be used or consumed” and “Use By” is used to “applies to perishable products that should be consumed by the date on the package and discarded after that date” (Grocers Manufacturers Association, 2019). The US FDA strongly supported the industry’s voluntary use of the “Best If Use By” introductory phrase for the quality-based food date labels but was not addressing the use of a “Use by” label for safety reasons (Food and Drug Administration, 2019). Other than the United States, most developed countries required food date labeling for most food products. The EU mandates the use of food date labeling for food products. Australia, New Zealand, and the EU clearly regulate the UBD related to food safety and “Best Before” related to food quality (Newsome et al., 2014).

To mitigate food waste, stakeholders need to well clarify and regulate the food date labeling, and customers need more information about fresh produce quality and about safety after the open date. The US Food and Drug Administration (2010) reported that the expected shelf-life for bagged fresh-cut leafy green is approximately 12–16 days after production at 4^°^C and the sensory quality of fresh leafy green can last at least a week after the open date labels (US Food and Drug Administration, 2010). However, limited study on microbial food quality after “open dates” has been reported. As fresh fruits and vegetables comprise the largest part of food waste in the food system (Augustin et al., 2020), this study presented bacterial RL quality on and after the UBD based on culture-dependent and culture-independent methods. The results showed that RL bacterial communities had no change in abundance, diversity, or composition from the UBD to UBD5. RL on the UBD5 had no increase of microbial quality or safety risk compared to RL on the UBD.

## Conclusion

In summary, this study determined there was no difference in bacterial abundance, diversity, and composition of bacterial communities in three brands of RL on the UBD5 compared with RL on the UBD, suggesting that the microbial quality of RL remained the same at two storage time points. Factors of “brand” and “harvesting season” played more significant roles in shaping the bacterial communities present in RL samples. The study provides first-hand information about the microbial quality of fresh produce on the UBD and a few days after the UBD.

## Data Availability Statement

The datasets presented in this study can be found in online repositories. The names of the repository/repositories and accession number(s) can be found in the article/Supplementary Material.

## Author Contributions

CL contributed to experimental design, experiment, manuscript writing, and data analysis. LW contributed to funding acquisition, experimental design, manuscript revising, and supervision. Both authors contributed to the article and approved the submitted version.

## Conflict of Interest

The authors declare that the research was conducted in the absence of any commercial or financial relationships that could be construed as a potential conflict of interest.

## Publisher’s Note

All claims expressed in this article are solely those of the authors and do not necessarily represent those of their affiliated organizations, or those of the publisher, the editors and the reviewers. Any product that may be evaluated in this article, or claim that may be made by its manufacturer, is not guaranteed or endorsed by the publisher.

## Footnotes

1
https://www.arb-silva.de/
