# Supplementary material for: The Microbial Quality of Commercial Chopped Romaine Lettuce Before and After the “Use By” Date
Source: Front Microbiol. 2022 Apr 11;13:850720. doi: 10.3389/fmicb.2022.850720 (PMC9036107; doi:10.3389/fmicb.2022.850720)
Supplement: Supplementary file 1 [file Image_1.pdf]

# *Supplementary Material*

## Supplementary Figures

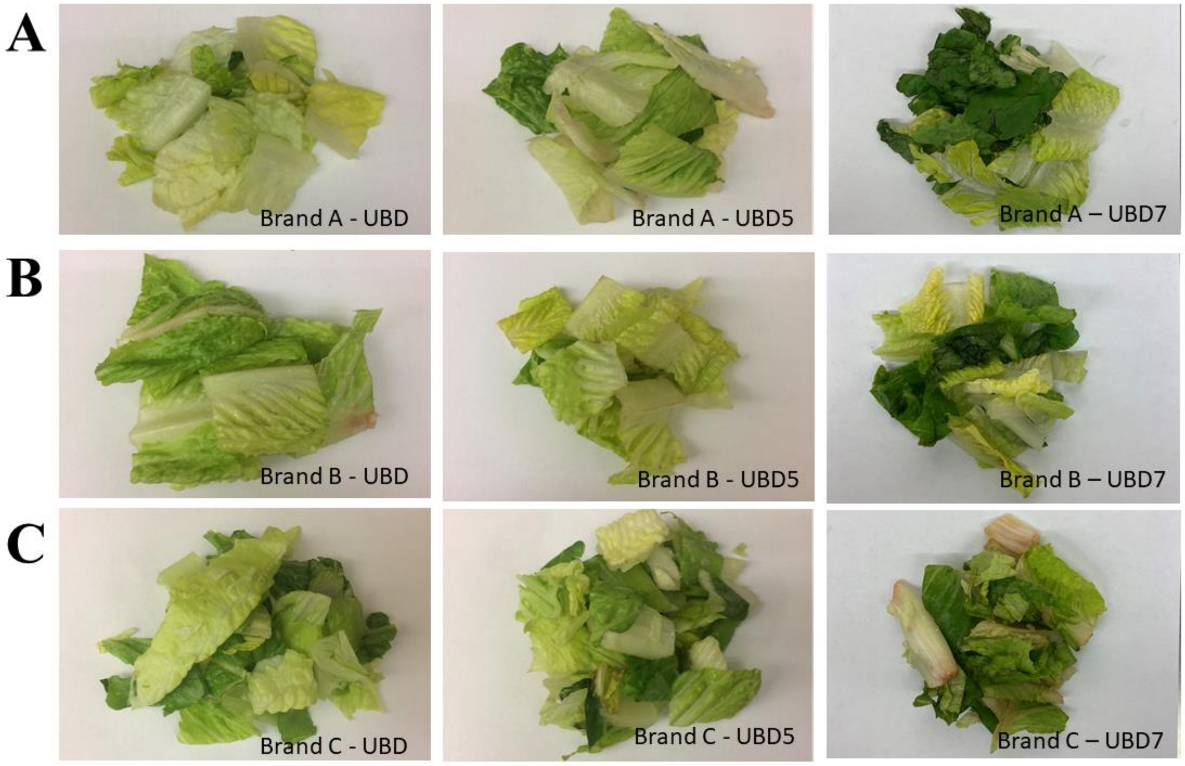


**Supplementary Figure 1.** Photos of three brands (A, B, and C) of RL on UBD (left), UBD5 (middle), and UBD7 (right). The UBD, UBD5, and UBD7 are abbreviations for the "Use By" date, 5 days after UBD, and 7 days after UBD, respectively.

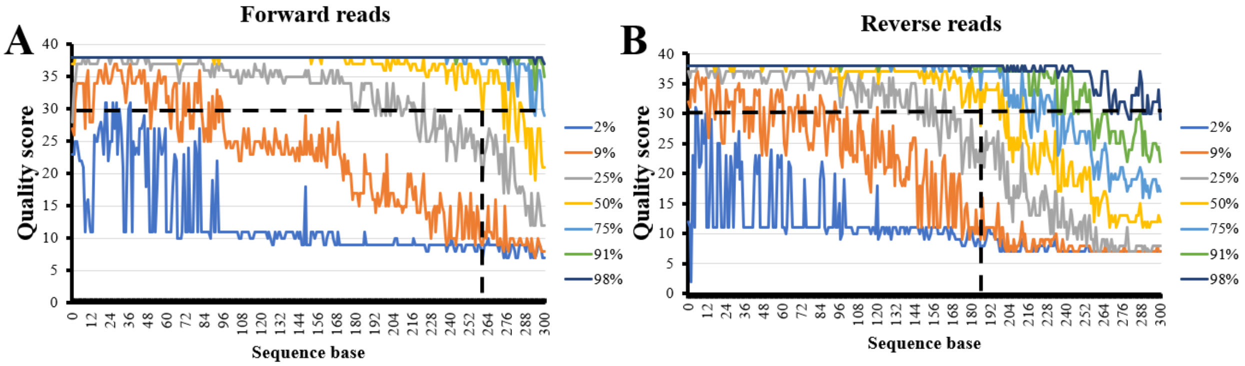


**Supplementary Figure 2.** Interactive quality plots of forward and reverse reads with 300 bases each. (A) Interactive quality plots of the forward read with 300 bases for seven percentiles. (B) Interactive quality plots of the reverse read with 300 bases for seven percentiles, including 2%, 9%, 25%, 50%, 75%, 91%, and 98%. The horizontal dash line represents the threshold of Phred quality score as 30 (Q30). The vertical dash line stands for the intersection points between Q30 dash line and the 50% interactive quality curve.

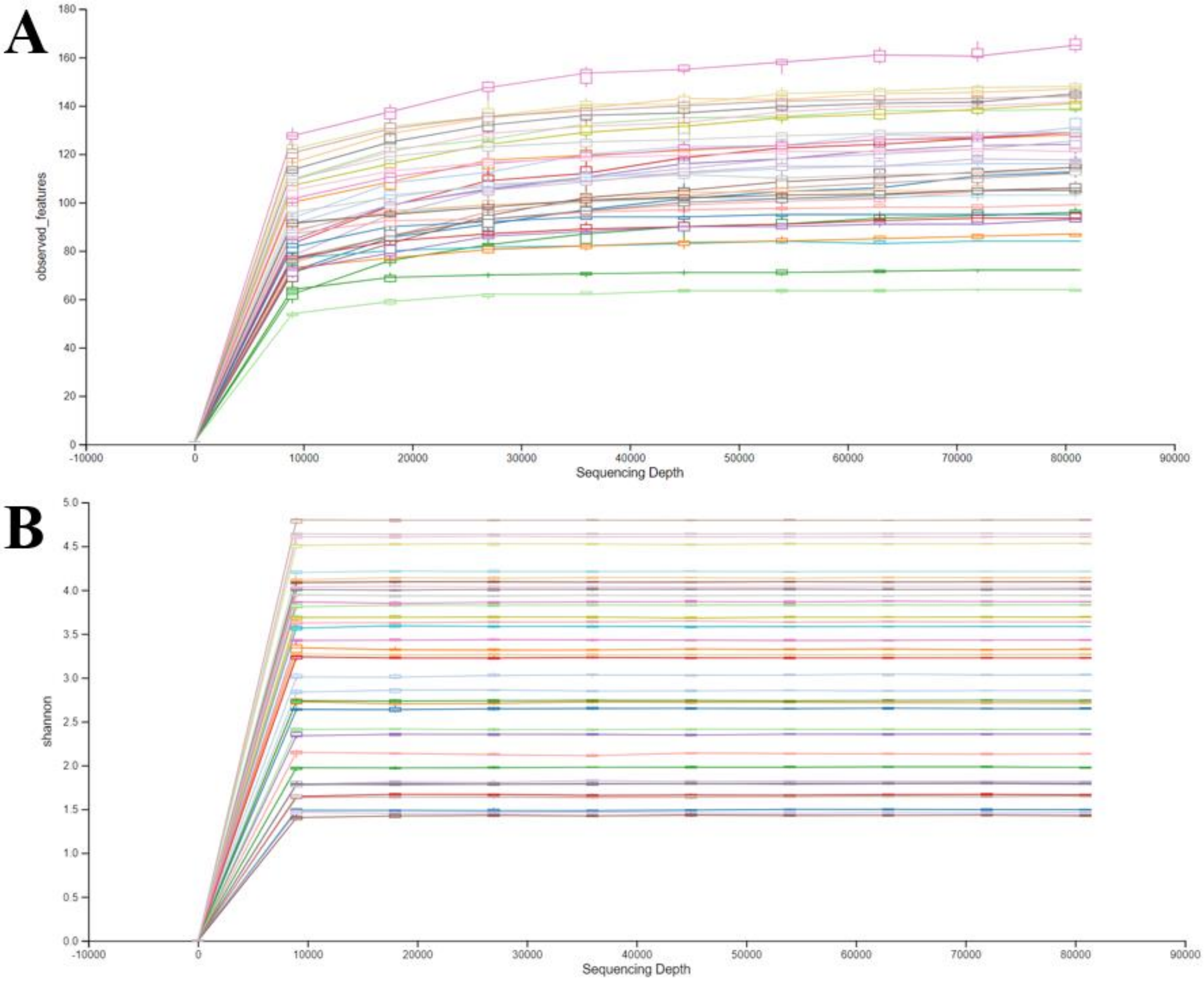


**Supplementary Figure 3.** Rarefication curves of alpha diversity as a function of sequencing depth (80,938 reads) of 36 samples. (A) Rarefication curves based on the observed features. (B) Rarefication curves based on the Shannon index.

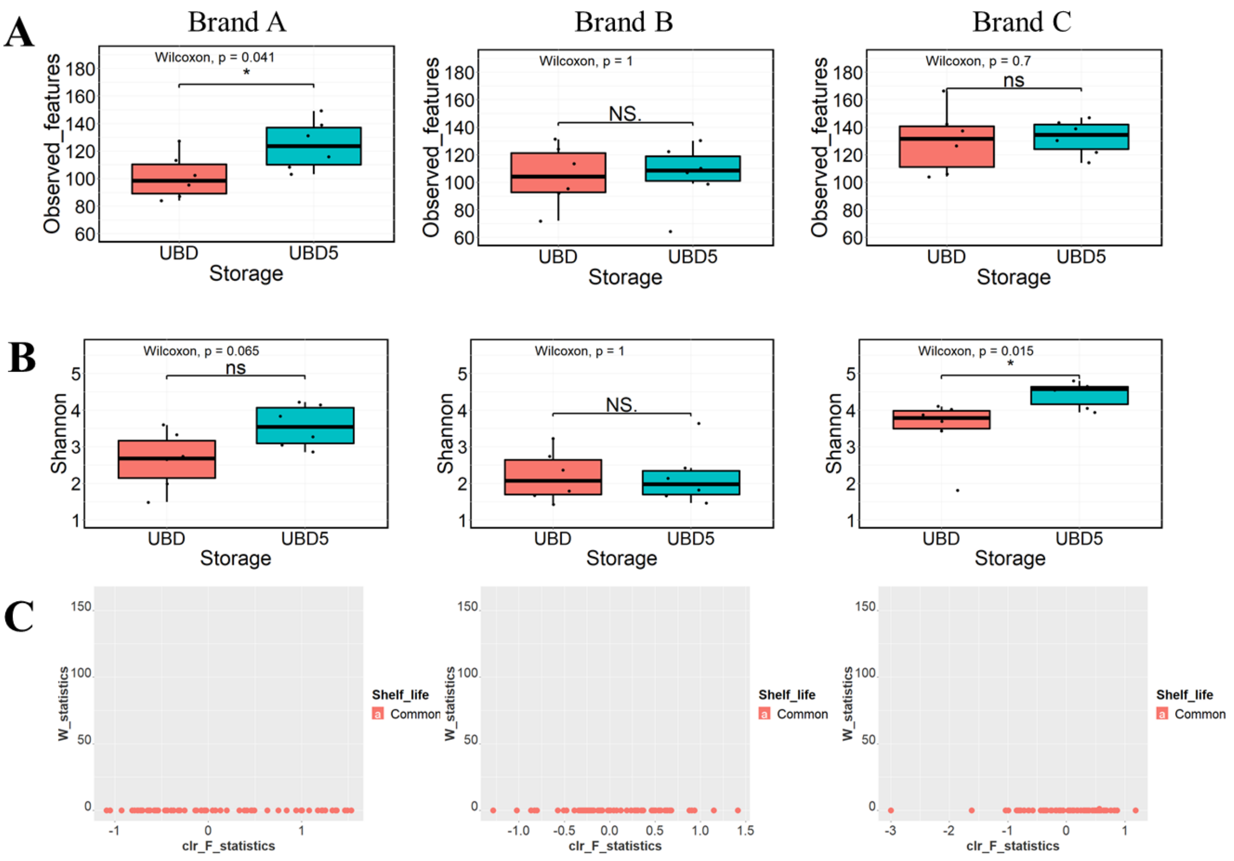


**Supplementary Figure 4.** The Alpha diversity variation based on the observed feature numbers and Shannon index values of bacterial communities in three brands (A, B, and C) on UBD and UBD5. (A) boxplots of observed feature numbers; (B) boxplots of Shannon index values; (C) Volcano plot of bacteria biomarkers for RL on UBD and UBD5 identified by the ANCOM test. UBD and UBD5 mean the "Use By" date and five days after the UBD. The Wilcoxon rank test was used for the pairwise comparison. The Kruskal-Wallis test was applied for the overall comparisons among three groups. "*" stands for $P < 0.05$. "NS" means no significance was observed between two groups. "Common" represents bacterium with no differential abundance between UBD and UBD5.
