# Supplementary material for: The Microbial Quality of Commercial Chopped Romaine Lettuce Before and After the “Use By” Date
Source: Front Microbiol. 2022 Apr 11;13:850720. doi: 10.3389/fmicb.2022.850720 (PMC9036107; doi:10.3389/fmicb.2022.850720)
Supplement: Supplementary file 2 [file Table_1.docx]

Supplementary Material

## Supplementary Tables

**Supplementary Table 1.** Statistical summary of actual sequence variants (ASVs) before and after denoising process during the QIIME 2 analysis.

|  | Number of samples | Total frequency | Minimum frequency | Median frequency | Maximum frequency |
| --- | --- | --- | --- | --- | --- |
| Sequences before denoising | 36 | 9,713,248 | 172,148 | 278,255 | 375,736 |
| Sequences after denoising | 36 | 6,051,758 | 80,938 | 172,073 | 250,847 |
