# Supplementary material for: The Microbial Quality of Commercial Chopped Romaine Lettuce Before and After the “Use By” Date
Source: Front Microbiol. 2022 Apr 11;13:850720. doi: 10.3389/fmicb.2022.850720 (PMC9036107; doi:10.3389/fmicb.2022.850720)
Supplement: Supplementary file 3 [file Data_Sheet_1.docx]

Supplementary Material

# Supplementary Data

The raw 16S rRNA gene sequencing data and metadata from this study have been submitted to GenBank Sequence Read Archive under the BioProject ID PRJNA792031 in NCBI.
